# W27 IgA suppresses growth of *Escherichia* in an in vitro model of the human intestinal microbiota

**DOI:** 10.1038/s41598-021-94210-8

**Published:** 2021-07-16

**Authors:** Kengo Sasaki, Tomoyuki Mori, Namiko Hoshi, Daisuke Sasaki, Jun Inoue, Reiko Shinkura, Akihiko Kondo

**Affiliations:** 1 Graduate School of Science, Technology and Innovation, Kobe University, 1-1 Rokkodai-cho, Nada-ku, Kobe, Hyogo 657-8501 Japan; 2 BioPalette Co., Ltd., 6-3-7 Minatojima Minamimachi, Chuo-ku, Kobe, Hyogo 650-0047 Japan; 3 Laboratory of Immunology and Infection Control, Institute for Quantitative Biosciences, The University of Tokyo, 1-1-1 Yayoi, Bunkyo-ku, Tokyo, 113-0032 Japan; 4 Division of Gastroenterology, Department of Internal Medicine, Graduate School of Medicine, Kobe University, 7-5-2 Kusunoki-cho, Chuo-ku, Kobe, Hyogo 650-0017 Japan; 5 Collaborative Research Institute for Innovative Microbiology, The University of Tokyo, 1-1-1 Yayoi, Bunkyo-ku, Tokyo, 113-0032 Japan; 6 Core Research for Evolutional Science and Technology, Japan Agency for Medical Research and Development, Tokyo, Japan; 7 RIKEN Center for Sustainable Resource Science, 1-7-22 Suehiro-cho, Tsurumi-ku, Yokohama, Kanagawa 230-0045 Japan

**Keywords:** Environmental biotechnology, Nutrition

## Abstract

W27 monoclonal immunoglobulin A (IgA) suppresses pathogenic *Escherichia coli* cell growth; however, its effect on the human intestine remains unclear. We aimed to determine how W27 IgA affects the human colonic microbiota using the in vitro microbiota model. This model was established using fecal samples collected from 12 healthy volunteers; after anaerobic cultivation, each model was found to retain the genera found in the original human fecal samples. After pre-incubating W27 IgA with the respective fecal sample under aerobic conditions, the mixture of W27 IgA (final concentration, 0.5 μg/mL) and each fecal sample was added to the in vitro microbiota model and cultured under anaerobic conditions. Next-generation sequencing of the bacterial 16S rRNA gene revealed that W27 IgA significantly decreased the relative abundance of bacteria related to the genus *Escherichia* in the model. Additionally, at a final concentration of 5 μg/mL, W27 IgA delayed growth in the pure culture of *Escherichia coli* isolated from human fecal samples. Our study thus revealed the suppressive effect of W27 IgA on the genus *Escherichia* at relatively low-concentrations and the usefulness of an in vitro microbiota model to evaluate the effect of IgA as a gut microbiota regulator.

## Introduction

The human gastrointestinal tract harbors a complex community of over trillions of microbial cells, which play a central role in human health^1^. Gut microbiota promotes food digestion and xenobiotic metabolism and regulates innate and adaptive immunological processes^2^. Many studies have shown that dysbiosis, defined as the persistent imbalance of gut microbiota, is associated with diseases such as inflammatory bowel disease, irritable bowel syndrome, diabetes, obesity, cancer, cardiovascular, and central nervous system disorders^2,3^. Manipulation of the intestinal microbiota using prebiotics, probiotics, and fecal microbiota transplants is an important strategy for disease prevention and treatment^4^.

Secretory immunoglobulin A (IgA) is the most abundant class of antibody isotypes found in the intestinal lumen^5^. Secretory IgA interacts with various intestinal antigens including self-antigens, food components, and intestinal microbiota^6^. Predominantly, IgA constitutes polyreactive specificities, mostly to coat commensal bacteria during homeostasis^7^. Other typical non-polyreactive IgA are predominantly triggered by pathogens and exhibit classical features of T cell-dependent, extensive somatic hypermutation and affinity maturation (generating high-affinity)^8^. Recently, Okai et al.^9,10^ demonstrated that a unique mouse monoclonal IgA antibody (clone W27) had high affinity for a variety of bacteria (showing a polyreactive nature) and suppressed the in vitro growth of *Escherichia coli* (*E. coli*) cells. Oral administration of W27 IgA prevented the development of dextran sodium sulfate-induced colitis in mice by modulating the intestinal microbiota to reduce genus *Escherichia*/*Shigella* and expand genus *Lactobacillus* (generally considered to be probiotic)^11^. These results have aroused much interest in the effect of W27 IgA on the human gut microbiota. However, to the best of our knowledge, studies on gut microbiota modulation by W27 IgA have so far been restricted to mice. Since the composition of the gut microbiota in mice is different from that in humans^12^, we aimed to study the effect of W27 IgA on the human gut microbiome and assess its therapeutic potential.

We previously reported an in vitro human colonic microbiota model (named as Kobe University human intestinal microbiota model, KUHIMM), which can capture most of the microbial species in a fecal sample^13^. Although in vitro models cannot provide the complete host factors, they are useful tools for studying and uncovering the microbiota response to different compounds^14^. Here, we examined the effect of W27 IgA on the human colonic microbiota using KUHIMM.

## Results

### W27 IgA suppresses genus *Escherichia* at low concentration

**Figure 1:**
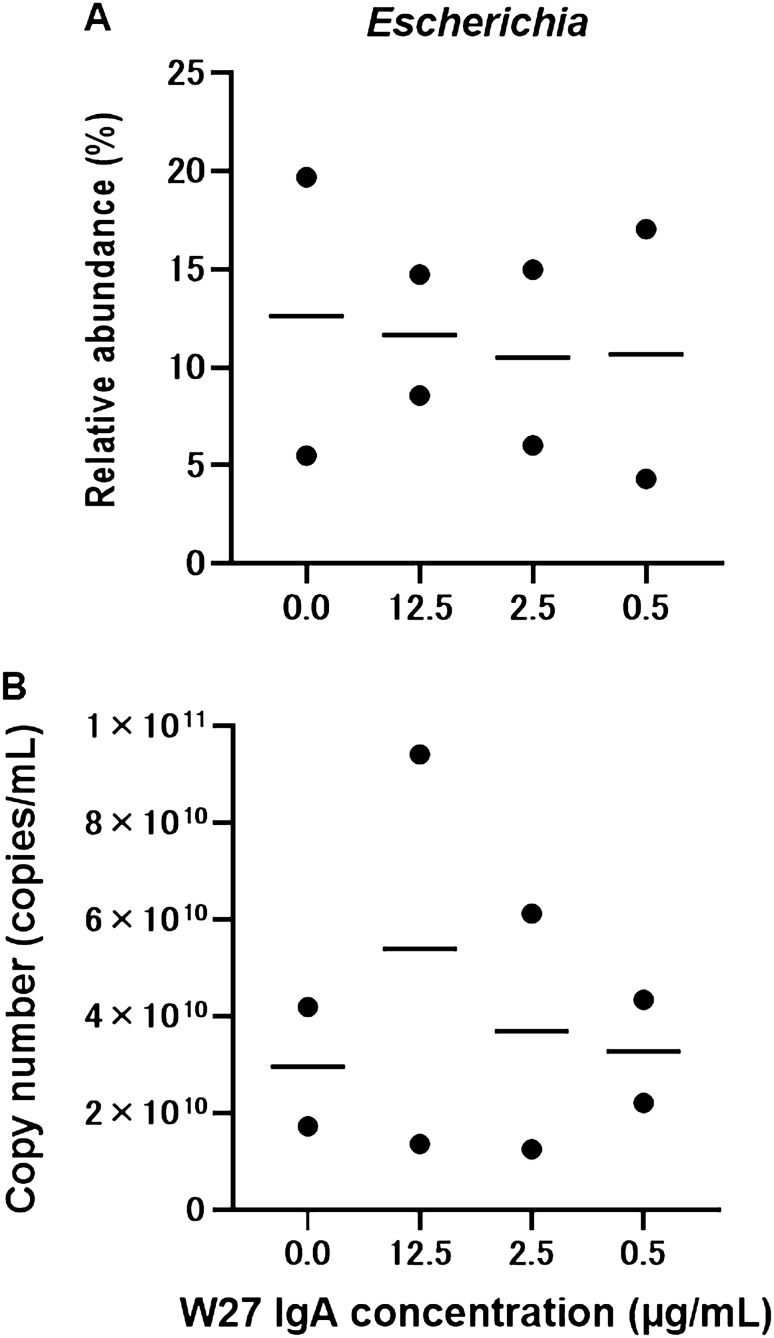
(**A**) Relative abundance and (**B**) absolute copy number of bacteria related to genus *Escherichia* in an in vitro human colonic microbiota model (KUHIMM) without (0.0 µg/mL) and with W27 IgA (12.5, 2.5, and 0.5 µg/mL) after 48 h of fermentation. The bars shown represent mean of two technical/biological replicates. *Escherichia* copy number = (Total copy number) × (Relative abundance).

To evaluate the effective W27 dose for human microbial culture, the optimal W27 IgA concentration was assessed using an in vitro human colonic microbiota model, KUHIMM. First, we preincubated one fecal sample (total 250 μL) with 200, 1000, or 5000 μg/mL W27 IgA under aerobic conditions at 37 °C for 3 h, in order to increase the efficacy of IgA binding. This concentration range is comparable with that of secretary IgA in the human stool (approximately 500–3000 μg/mL)^15^. This mixture was then transferred to the model culture system to establish KUHIMM, in which the final concentration of W27 IgA was 0, 0.5, 2.5, or 12.5 μg/mL in duplicate experiments. Total bacterial DNA was extracted from the original fecal inoculum and the corresponding KUHIMM cultures after 48 h of fermentation. In all 4 KUHIMM samples, eubacterial copy numbers, evaluated by quantitative real-time PCR, reached (5.34 ± 4.76) × 10^11^ copies/mL in the fecal inoculum (4.68 × 10^8^ copies/mL) (Supplementary Fig. S1). Next-generation sequencing was performed using the Illumina MiSeq system to analyze the V3–V4 region of bacterial 16S rRNA gene sequences in the fecal inoculum and the corresponding KUHIMM cultures. The relative abundance and absolute copy number of bacteria related to the genus *Escherichia* were found to have a tendency to decrease with W27 IgA at 0.5 and 2.5 μg/mL, compared with W27 IgA at 12.5 μg/mL (Fig. 1). A lower concentration of W27 IgA was found more effective at suppressing the genus *Escherichia* in KUHIMM. Thus, W27 IgA was applied to subsequent KUHIMM experiments at final concentration of 0.5 μg/mL.

### At final concentration of 0.5 μg/mL, W27 IgA suppresses genus *Escherichia* in KUHIMMs

KUHIMMs were established using the 12 human fecal samples as inoculums. Each fecal sample was preincubated with 200 μg/mL of W27 IgA or mouse monoclonal IgA, which has low specificity for microorganisms, as control under aerobic conditions at 37 °C for 3 h. Each fecal sample was also prepared and incubated for 3 h without W27 IgA. Then, to examine the effect of IgA on the microbiota, particularly on bacteria related to the genus *Escherichia*, we added each of these fecal samples to the model culture system. The final concentration of W27 IgA or mouse monoclonal IgA in KUHIMM was 0.5 μg/mL. DNA was extracted from the fecal inoculums and corresponding KUHIMM cultures without IgA (control) and with W27 IgA (+ W27 IgA) or mouse monoclonal IgA (+ Mouse IgA) after 48 h of fermentation. In all KUHIMM samples, the eubacterial copy numbers reached 1.44 to 5.53 × 10^11^ copies/mL (Supplementary Fig. S2).

Next-generation sequencing obtained a total of 1,213,033 quality reads from the 12 fecal samples and the corresponding KUHIMMs with and without IgA (Table 1). The number of operational taxonomic units (OTUs) was lower in the KUHIMMs compared to the original fecal samples (Wilcoxon signed-rank test, *p* = 0.0005). However, there was no significant difference in the number of OTUs between KUHIMMs without IgA and with IgA (Wilcoxon signed-rank test, *p* > 0.05). The other alpha diversity measures (Shannon index, and Faith’s phylogenetic diversity) were also lower in the KUHIMMs than in the original fecal samples (Wilcoxon signed-rank test, *p* < 0.05); however, there was no significant difference between KUHIMMs without IgA and with IgA (Wilcoxon signed-rank test, *p* > 0.05). Thus, the microbial richness and diversity in the KUHIMMs did not change with the addition of W27 IgA or mouse monoclonal IgA at 0.5 μg/mL.

**Table 1:** Summary of 16S rRNA gene sequencing data and α-diversity values [Shannon index and Faith’s phylogenetic diversity (PD)].

	Feces	KUHIMM		
		Control	+ W27 IgA	+ Mouse IgA
Read counts	22,340 ± 3,797	26,063 ± 9,806	27,090 ± 5,774	25,593 ± 6,594
Observed OTUs	380.2 ± 113.7	215.2 ± 74.7*	210.0 ± 56.8	213.0 ± 46.0
Shannon index	7.72 ± 0.54	6.28 ± 0.46*	6.32 ± 0.46	6.35 ± 0.45
Faith’s PD	17.5 ± 5.2	10.6 ± 2.8*	11.7 ± 3.0	10.7 ± 1.4

In addition to twelve human fecal samples (Feces), the corresponding cultures without IgA (Control), the corresponding cultures with W27 IgA (+ W27 IgA), and the corresponding cultures with mouse monoclonal IgA (+ Mouse IgA) were sampled after 48 h of fermentation. The values are represented as mean ± standard deviation.

Asterisks (*) represent significant differences (*p < 0.05) (n = 12) between the microbiota in the original feces and in corresponding cultures without IgA using Wilcoxon signed rank test. Significant differences were not detected between KUHIMMs without and with IgA.

**Figure 2:**
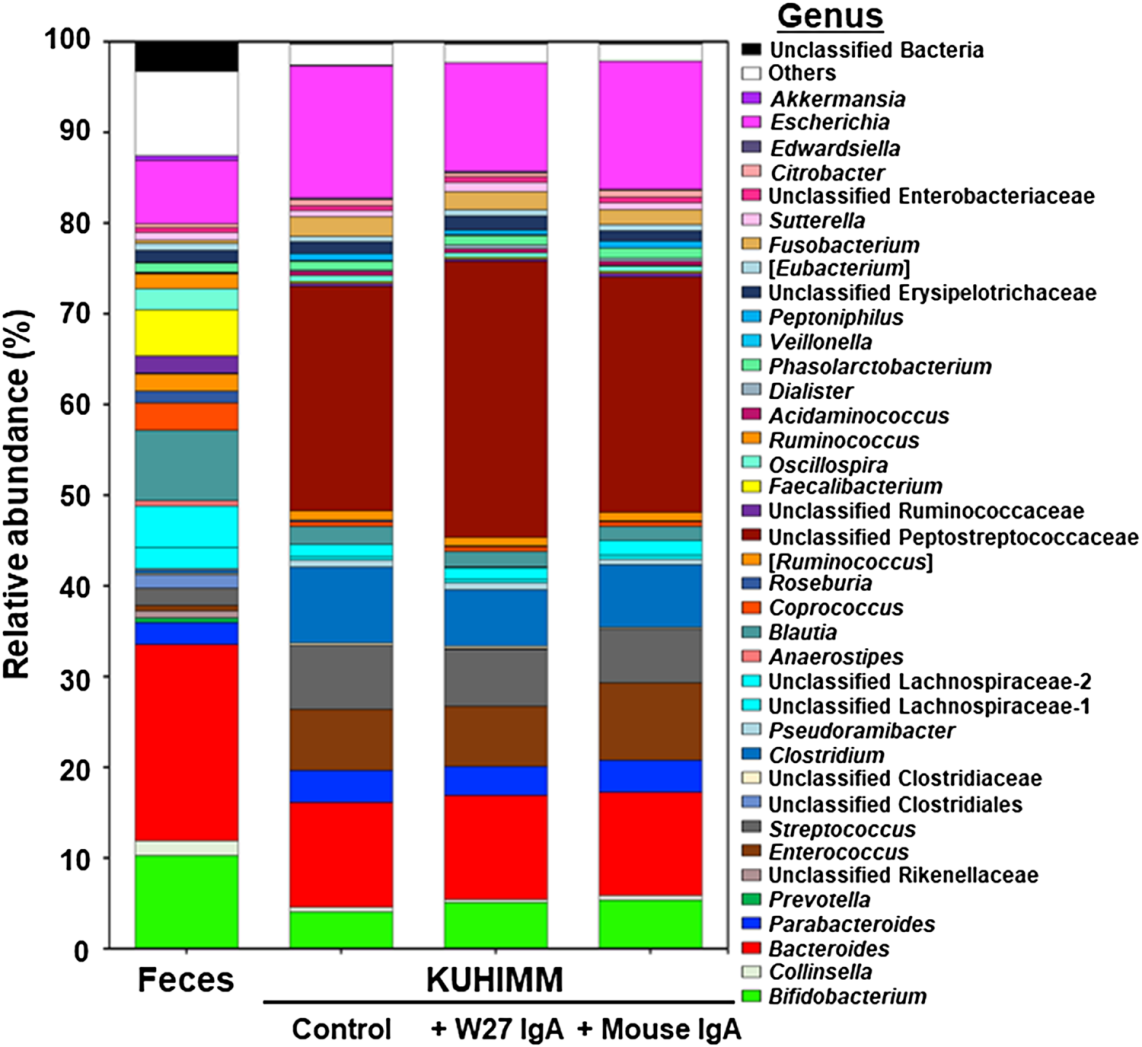
Genus-level compositional views of bacteria in twelve human samples. The means are shown. Original fecal suspensions (Feces; n = 12), and the corresponding KUHIMM cultures (sampled after 48 h of fermentation): without IgA (Control), with W27 IgA (+ W27 IgA), and with mouse monoclonal IgA (+ Mouse IgA) were analyzed. Genera with lower abundance (< 1.0%) and lower similarity (< 97%) were included as “Others” and “Unclassified Bacteria” respectively.

**Figure 3:**
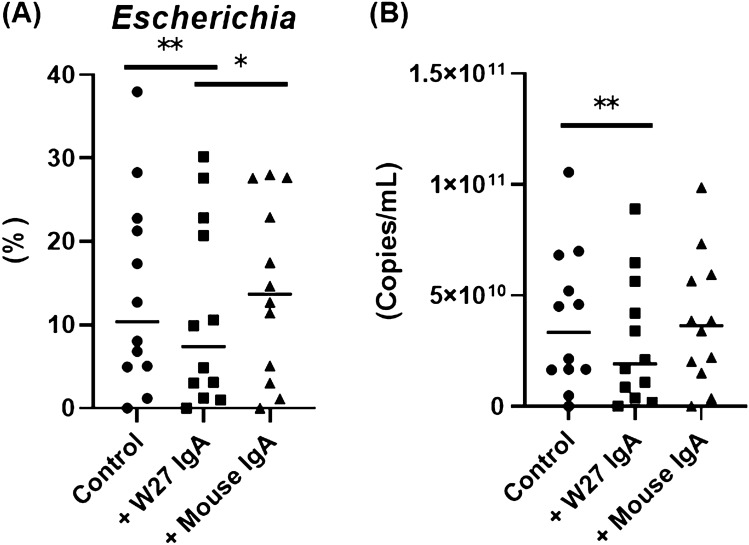
The relative abundance distributions (**A**) and absolute copy numbers (**B**) of genus *Escherichia* in KUHIMM (n = 12). The distribution for each group is shown (Control, + W27 IgA, and + Mouse IgA). The bars represent mean of twelve technical/biological replicates. *Escherichia* copy number = (Total copy number) × (Relative abundance). **p < 0.01, and *p < 0.05, Wilcoxon signed-rank test.

For most of the KUHIMM cultures (11 of 12), the addition of IgA did not affect the structure of the microbiota (Supplementary Fig. S3). Bacterial genus-level compositional analyses of the microbiota in the feces, control, + W27 IgA, and + mouse IgA are shown in Fig. 2. Almost all bacterial genera in the original feces were also detected in the corresponding KUHIMMs. The most significant decrease in the relative abundance of the genus *Escherichia* was observed in KUHIMMs with W27 IgA (+ W27 IgA), compared to those without IgA (Control) and those with mouse monoclonal IgA (Wilcoxon signed-rank test, *p* = 0.002 and 0.02, respectively); however, no significant difference was observed between + mouse IgA and control (Wilcoxon signed-rank test, *p* = 0.90) (Fig. 3A). In addition, KUHIMMs with W27 IgA (+ W27 IgA) also exhibited a decrease in the absolute number of the members of the genus *Escherichia*, compared to those without IgA (Control) and those with mouse monoclonal IgA (Wilcoxon signed-rank test, *p* = 0.002 and 0.05, respectively) (Fig. 3B). For almost all other genera, no major difference was observed between + mouse IgA and control (Supplementary Table S1). In contrast, administration of W27 IgA increased the relative abundance of bacteria related to the *Suttrella* and *Bifidobacterium* genera. Thus, addition of 0.5 μg/mL W27 IgA selectively decreased the bacteria related to genus *Escherichia*.

### W27 IgA suppresses *Escherichia coli* isolated from human feces

**Figure 4:**
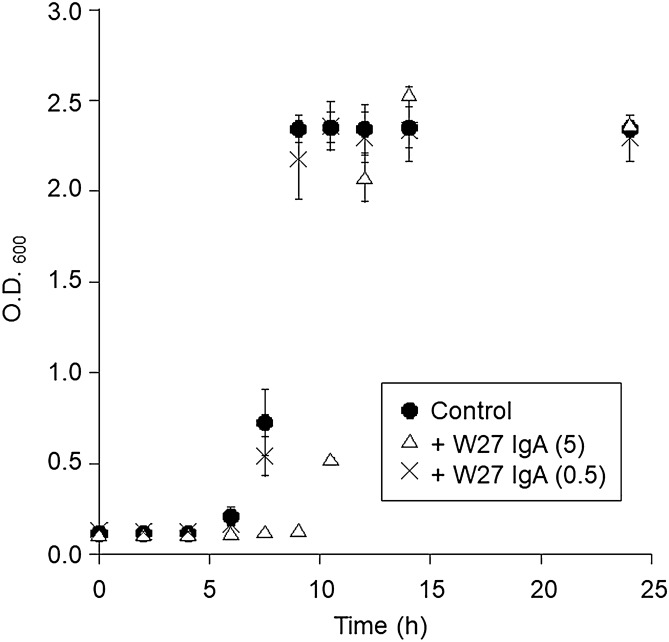
Growth of isolated *Escherichia coli* subcultured in Gifu Anaerobic Medium with 5.0 or 0.5 µg/mL W27 IgA [+ W27 IgA (5) or + W27 IgA (0.5)] or without IgA (Control). Growth is represented as the OD_600_. Data are shown as means ± standard deviation (n = 3).

We investigated the effect of W27 IgA on *E. coli* isolated from a human fecal sample, using nutrient broth with 0.5% NaCl under aerobic conditions. Isolated *E. coli* (800 cells/mL) was preincubated with or without W27 IgA (200 or 20 μg/mL) at 37 °C for 1 h under aerobic conditions. We then added isolated *E. coli* or the mixture with W27 IgA to Gifu Anaerobic Medium under anaerobic conditions and incubated at 37 °C for 24 h. The final concentration of W27 IgA was 5.0 or 0.5 μg/mL. As indicated by the optical density at 600 nm (OD_600_), W27 IgA delayed the growth of isolated *E. coli* at a final concentration of 5.0 μg/mL (Fig. 4). However, treatment with 0.5 μg/mL W27 IgA had no significant effect on the growth of *E. coli* as compared to untreated controls. Final microbial concentrations were similar between isolated *E. coli* with (5.0 or 0.5 μg/mL) and without (0 μg/mL) W27 IgA after 24 h of incubation.

## Discussion

W27 IgA at a final concentration of 0.5 μg/mL specifically suppressed bacteria related to the genus *Escherichia* in the in vitro human colonic microbiota model, KUHIMM, which harbored complex microbiota at more than 10^11^ copies/mL. This microbial number was comparable to the reported cell densities in the human colon (approximately 10^11^ cells/mL)^16^. However, higher concentrations of W27 IgA were less effective in suppressing the growth of *Escherichia*. Poly-reactive W27 IgA might react with various kinds of microorganisms, and might not be specific to *Escherichia* at high concentrations. Our results suggest that W27 IgA exhibited high specificity toward the genus *Escherichia* at relatively low concentrations. This result corresponds to previous data showing that W27 IgA specifically recognized the metabolic enzyme, serine hydroxymethyltransferase, from *E. coli*, rather than that from *Bifidobacterium bifidum*, *Blautia coccoides*, and *Lactobacillus casei*^9^. To our knowledge, this is the first report on the influence of W27 IgA on the human colonic microbiota model. In addition, W27 IgA at a final concentration of 5 μg/mL suppressed isolated *E. coli* in an in vitro model harboring *E. coli* alone. The final concentration of W27 IgA in the *E. coli* model was ten times higher than that in the KUHIMMs. The relative abundance of the *Enterobacteriaceae* family, including the *Escherichia* genus, was found to be approximately 2% in the colon of 13 healthy human subjects^17^. Thus, 0.5 μg/mL of W27 IgA as a daily dose would be sufficient to suppress the growth of the *Escherichia* genus in healthy human subjects. This concentration of IgA (0.5 μg/mL) corresponds to 200 μg of IgA administered into the human colon, as the human colon content has been estimated at approximately 400 mL^16^. In contrast, the relative abundance of the *Enterobacteriaceae* family was found to have been increased to an average of approximately 24% (12 times higher than that in healthy subjects) in the colon of 13 patients with ulcerative colitis^17^. Several studies have demonstrated that proliferation of *E. coli* may influence the inflammatory process in the gastrointestinal tract^18^. Therefore, suppression of *E. coli* is important to prevent the progression of inflammation. Thus, patients with ulcerative colitis might require 6 (= 0.5 × 12) μg/mL of W27 IgA, corresponding to 2.4 mg/day, to suppress *E. coli*. In contrast, W27 IgA increased relative abundance of bacteria related to genus *Bifidobacterium*, which are probiotic. This result corresponded with a previous report indicating that lower family *Bifidobacteriaceae* (including genus *Bifidobacterium*) and higher family *Enterobacteriaceae* (including genus *Escherichia*) in the gut of elderly individuals exhibit low IgA response, compared to those in the gut of younger adults^19^. These results indicate that W27 IgA and intestinal IgA might have similar role in suppressing potentially pathogenic microbes and supporting beneficial microbes.

To the best of our knowledge, this is the first study to investigate the role of IgA using an in vitro model mimicking the human colonic microbiota. We utilized an in vitro batch fermentation system that is fast, easy to operate, and reproducible^20^. The limitation of KUHIMM is that it utilizes simple batch fermentation for 48 h; therefore, the long-term-effective dose of IgA could not be evaluated. An increase in the relative abundance of *Lachnospiraceae* was not observed in the KUHIMM upon addition of W27 IgA, whereas previous study reported that *Lachnospiraceae* abundance was increased in mice supplemented with W27 IgA for 4 weeks^9^. In addition, we observed differences in the composition of microbiota between KUHIMM and fecal samples, such as an increase in unclassified *Peptostreptococcaceae*, *Streptococcus*, and *Enterococcus* in the KUHIMM culture. Further improvement of KUHIMM to address these limitations is currently underway. In addition, further investigation of W27 IgA at different concentrations and different treatment durations is needed to elucidate the mechanism by which it alters the composition of gut microbiota.

In conclusion, these results demonstrate that KUHIMM is useful for simulating the effect of therapeutic IgA as a gut microbial regulator in human patients. Further, we confirmed that W27 IgA, at a relatively low concentration (0.5 μg/mL), can suppress *Escherichia* growth in vitro in KUHIMM harboring complex human microbial species, indicating its potential for treating intestinal diseases with a disturbed balance of *Escherichia* species.

## Methods

### Fecal specimen collection

Fecal samples were obtained from 12 healthy Japanese volunteers, who had not been treated with antibiotics for more than 6 months prior to the experiment. All participants were recruited according to the following inclusion criteria: age 20–60 years, Japanese, non-smoking, and with good health and physical condition. All subjects provided written informed consent prior to specimen collection. Immediately after collection, each fecal sample was stored in an anaerobic culture swab (212,550 BD BBL Culture Swab; Becton, Dickinson and Company, New Jersey, USA) and used within 24 h. The study was performed in accordance with the guidelines of Kobe University Hospital, and was approved by the Institutional Ethics Review Board of Kobe University. All methods used in this study were in accordance with the principles of the Declaration of Helsinki.

### Operation of the model culture system with and without IgA

We used a small-scale multi-channel fermenter (Bio Jr. 8; ABLE, Tokyo, Japan) comprising eight parallel and independent anaerobic culturing vessels, as described by Sasaki et al.^13^. Each vessel contained autoclaved Gifu anaerobic medium (GAM [Code 05422]; Nissui Pharmaceutical Co., Ltd., Tokyo, Japan), with the initial pH adjusted to 6.5. The anaerobic conditions in the vessel were achieved by purging with a mixture of N_2_ and CO_2_ (80:20, 15 mL/min), which was filter-sterilized through a 0.2-μm polytetrafluoroethylene membrane filter (Pall Corporation, Port Washington, Ny, USA) at 37 °C for 1 h prior to cultivation. To prepare the fecal suspension, the fecal sample in the swab was suspended in phosphate buffer (0.1 M, 2.0 mL, pH 6.5, comprising of mixture of NaH_2_PO_4_ and Na_2_HPO_4_ at 61.65:28.35) supplemented with L-ascorbic acid (1.0% w/v; Wako Pure Chemical Industries, Osaka, Japan) in aerobic conditions.

W27 IgA was prepared as described previously^9^. Mouse IgA-LE/AF was purchased from Southern Biotechnologies (0106-14). The fecal suspension was preincubated with or without IgA at 37 °C under aerobic condition for 3 h. Cultivation in the fermentation jar was initiated by inoculating one fecal suspension with or without IgA (approximately 250 μL) into each vessel. During fermentation at 37 °C, the culture broth was stirred at 300 rpm with a magnetic stirrer and continuously purged with a filter-sterilized gas mixture. Aliquots of the culture broth were collected from the vessel 48 h after initiating the culture. The fecal suspensions and culture broth samples were then stored at − 20 °C until use. At first, adequate concentration of W27 IgA (0, 0.5, 2.5, or 12.5 μg/mL) was checked by aligning the pH conditions (6.4 or 6.8) at the end of operation.

### Profiling of bacterial 16S rRNA

Microbial genomic DNA was extracted from the fecal suspension and culture broth obtained from KUHIMM, as described previously^13^. Purified DNA was eluted into TE buffer (10 mM Tris–HCl, 1.0 mM EDTA) and stored at − 20 °C until use. Bacterial 16S rRNA genes (V3‒V4 region) were amplified using genomic DNA as the template with the primers S-D-Bact-0341-b-S-17 (5′-CCTACGGGNGGCWGCAG-3′) and S-D-Bact-0785-a-A-21 (5′-GACTACHVGGGTATCTAATCC-3′)^21^. PCR and amplicon pool preparation were performed according to the manufacturer’s instructions (Illumina, San Diego, CA, USA). PCR amplicons were purified using AMPure XP DNA purification beads (Beckman Coulter, Brea, CA, USA) and were eluted in 25 μL of 10 mM Tris (pH 8.5). Purified amplicons were quantified using the Agilent Bioanalyzer 2100 with DNA 1000 chips (Agilent Technology, Santa Clara, CA, USA) and the Qubit 2.0 instrument (Thermo Fisher Inc., Waltham, MA, USA), and were pooled at equimolar concentrations (5 nM). The 16S rRNA genes and an internal control (PhiX control v3; Illumina) were subjected to paired-end sequencing using the MiSeq instrument (Illumina) and the MiSeq Reagent Kit v3 (600 cycles; Illumina). The PhiX sequences were removed, and paired-end reads with Q scores ≥ 20 were joined using the Automated CASAVA 1.8 paired-end demultiplexed fastq, which was performed with the FASTQ Generation program on the Illumina Basespace Sequence Hub (https://basespace.illumina.com/). Sequence quality control and feature table construction of the sequence data were performed and corrected using QIIME 2 version 2020.8 (https://qiime2.org)^22^ and the DADA2 pipeline^23^. The taxonomic compositions of the OTUs were classified using the naive Bayes classifier trained on the Greengenes 13_8 99% OTU full-length sequence database (https://data.qiime2.org/2020.8/common/gg-13-8-99-nb-classifier.qza). The OTU data were then used for α-diversity estimation of Faith’s phylogenetic diversity^24^ and Shannon’s indices^25,26^.

### Real-time PCR analysis

Real-time PCR was performed to quantify total bacteria, using the LightCycler 96 system (Roche, Basal, Switzerland) with a universal primer set (5′-ACTCCTACGGGAGGCAGCAGT-3′ and 5′-GTATTACCGCGGCTGCTGGCAC-3′) targeting eubacteria^27^. PCR amplification was performed as described previously^28^.

### Isolation of *E. coli* from human feces

Fresh fecal samples derived from one human volunteer were prepared and cultured in Gifu anaerobic medium as described above. The human fecal fermentation culture was plated on the surface of autoclaved nutrient broth agar with 0.5% NaCl (composition per liter was 15 g agar, 5 g Bacto peptone, 3 g beef extract, and 5 g NaCl). The agar plate was incubated at 37 °C under aerobic conditions for 1 d. A single colony was picked, subcultured in nutrient broth medium with 0.5% NaCl, and then stored as the stock culture at − 80 °C after adding glycerol (20% [vol/vol]). Genomic DNA was extracted from the 24-h culture in each culture medium as described previously.

### Growth assay of isolated *E. coli*

Isolated *E. coli* were pre-cultured overnight in nutrient broth medium with 0.5% NaCl at 37 °C under aerobic conditions. The culture was then diluted to 800 cells/mL in phosphate-buffered saline and preincubated with or without 200 or 20 μg/mL of W27 IgA at 37 °C under aerobic conditions for 1 h. Then, *E. coli* with or without W27 IgA (final concentration: 5 or 0.5 μg/mL) was cultured in Gifu anaerobic medium at 37 °C under anaerobic conditions (N_2_: 80%, H_2_: 10%, CO_2_: 10%) for 24 h. Finally, the OD_600_ was measured using a spectrophotometer (UVmini-1240; Shimadzu, Japan).

### Statistical analysis

Data were compared between groups using the Wilcoxon signed-rank test or one-way ANOVA in JMP version 12 or GraphPad Prism software (GraphPad Prism 8), respectively. Statistical significance was set at *p* < 0.05.

## Supplementary Information

Supplementary Information.

## Data Availability

All sequences from the original fecal samples and corresponding KUHIMMs were deposited in MG-RAST as “Model Culture System of Human Colonic Microbiota IgA” under accession number mgm4922092.3-mgm4922148.3.
