# Supplementary material for: W27 IgA suppresses growth of Escherichia in an in vitro model of the human intestinal microbiota
Source: Sci Rep. 2021 Jul 16;11:14627. doi: 10.1038/s41598-021-94210-8 (PMC8285510; doi:10.1038/s41598-021-94210-8)
Supplement: Supplementary file 1 — Supplementary Information. [file 41598_2021_94210_MOESM1_ESM.pdf]

Supplementary Information

Title

W27 IgA suppresses growth of *Escherichia* in an *in vitro* model of the human intestinal microbiota

Authors

Kengo Sasaki[1,2,*], Tomoyuki Mori[3], Namiko Hoshi[4], Daisuke Sasaki[1], Jun Inoue[4], Reiko Shinkura[3,5,6], and Akihiko Kondo[1,7]

[1] Graduate School of Science, Technology and Innovation, Kobe University, 1-1 Rokkodai-cho, Nada-ku, Kobe, Hyogo 657-8501, Japan

[2] BioPalette Co., Ltd., 6-3-7 Minatojima Minamimachi, Chuo-ku, Kobe, Hyogo 650-0047, Japan

[3] Laboratory of Immunology and Infection Control, Institute for Quantitative Biosciences, The University of Tokyo, 1-1-1 Yayoi, Bunkyo-ku, Tokyo 113-0032, Japan

[4] Division of Gastroenterology, Department of Internal Medicine, Graduate School of Medicine, Kobe University, 7-5-2 Kusunoki-cho, Chuo-ku, Kobe, Hyogo 650-0017, Japan

[5] Collaborative Research Institute for Innovative Microbiology, The University of Tokyo, 1-1-1 Yayoi, Bunkyo-ku, Tokyo 113-0032, Japan

[6] Core Research for Evolutional Science and Technology, Japan Agency for Medical Research and Development, Tokyo, Japan.

[7] RIKEN Center for Sustainable Resource Science, 1-7-22 Suehiro-cho, Tsurumi-ku, Yokohama, Kanagawa 230-0045, Japan

*Corresponding author

Kengo Sasaki

Graduate School of Science, Technology and Innovation, Kobe University, 1-1 Rokkodai-cho, Nada-ku, Kobe, Hyogo 657-8501, Japan

+81-78-803-6196 (TEL/FAX)

sikengo@people.kobe-u.ac.jp

BioPalette Co., Ltd., 6-3-7 Minatojima Minamimachi, Chuo-ku, Kobe, Hyogo 650-0047, Japan

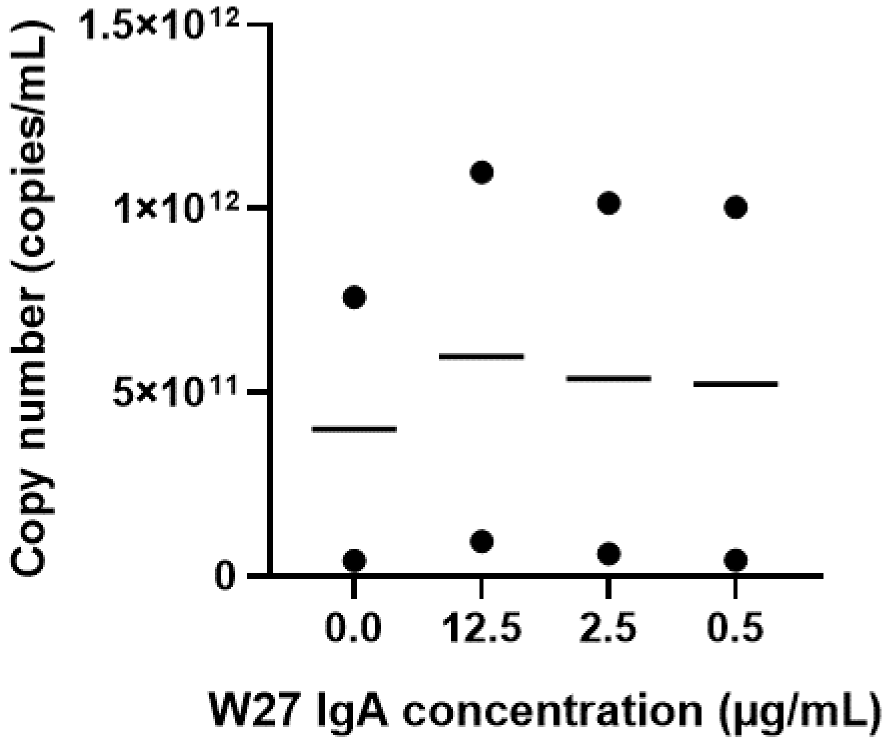

Supplementary Figure S1

Supplementary Figure S1. Absolute copy number of total bacteria in an *in vitro* human colonic microbiota model (KUHIMM) without (0.0 µg/mL) and with W27 IgA (12.5, 2.5, and 0.5 µg/mL) after 48 h of fermentation. The bars represent mean of two technical/biological replicates.

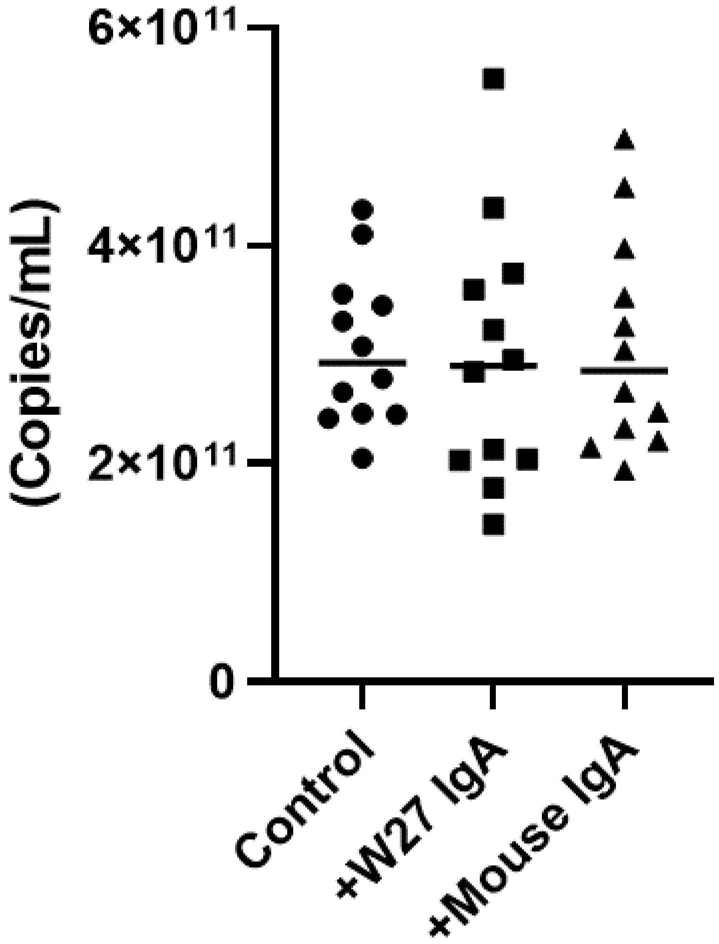

Supplementary Figure S2

Supplementary Figure S2. Absolute copy number of total bacteria in an *in vitro* human colonic microbiota model (KUHIMM) after 48 h of fermentation. The bars represent mean of twelve technical/biological replicates for each group (Control, +W27 IgA, and +Mouse IgA).

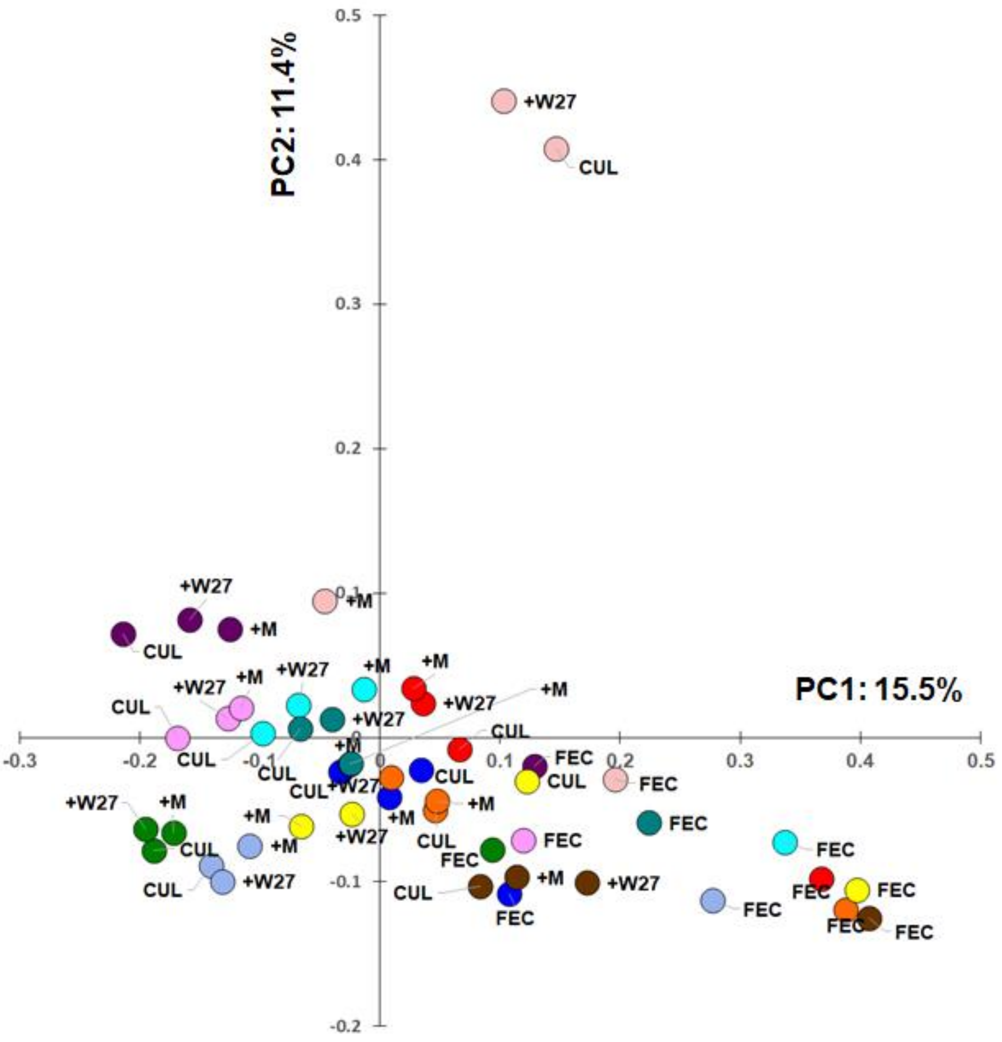

Supplementary Figure S3

Supplementary Figure S3. Clustering of microbiota with principal coordinate analysis using UniFrac. FEC: fecal inoculum; CUL: *in vitro* human colonic microbiota model (KUHIMM) culture without IgA after 48 h of fermentation; +W27: KUHIMM culture with W27 IgA; +M: KUHIMM culture with mouse monoclonal IgA. Different color represent different human samples. One-way ANOVA revealed that the differences in distances of FEC-CUL, FEC-+W27, and FEC-+M were not statistically significant (p = 0.32).

Supplementary Table S1. Microbiota composition in an *in vitro* human colonic microbiota model (KUHIMM).

Genus	Average ± Standard deviation			*P* value		
	Control	+W27 IgA	+Mouse IgA		Control vs. +W27 IgA	Control vs. +Mouse IgA
Akkermansia	0.019 ± 0.065	0.012 ± 0.040	0.029 ± 0.070	0.2679	>0.9999	0.6750
Escherichia	13.87 ± 11.80	11.25 ± 11.12	14.31 ± 10.58	0.0422	0.0865	>0.9999
Citrobacter	0.77 ± 1.63	0.47 ± 0.92	0.81 ± 1.66	0.2583	0.3745	>0.9999
Unclassified Enterobacteriaceae	0.56 ± 1.23	0.66 ± 1.62	0.70 ± 1.43	0.3641	0.6381	0.3460
Sutterella	0.70 ± 0.79	1.13 ± 1.28	0.82 ± 0.91	0.0323	0.0230	0.8725
Fusobacterium	1.75 ± 2.48	1.51 ± 2.52	1.34 ± 2.93	0.6719	>0.9999	0.7619
[Eubacterium]	0.62 ± 0.81	0.67 ± 0.58	0.63 ± 0.43	0.9372	>0.9999	>0.9999
Unclassified Erysipelotrichaceae	1.33 ± 1.32	1.58 ± 1.28	1.14 ± 0.95	0.3381	0.8016	>0.9999
Peptoniphilus	0.93 ± 1.30	0.64 ± 0.95	0.81 ± 1.22	0.6230	0.6774	>0.9999

Phascolactobacterium	0.89 ± 0.69	0.96 ± 0.58	1.09 ± 0.74	0.2381	>0.9999	0.1934
Dialister	0.16 ± 0.29	0.45 ± 1.07	0.39 ± 0.92	0.4803	0.5114	0.7229
Acidaminococcus	0.49 ± 1.10	0.49 ± 1.21	0.51 ± 1.19	0.9897	>0.9999	>0.9999
Ruminococcus	0.058 ± 0.063	0.045 ± 0.078	0.080 ± 0.095	0.3718	>0.9999	0.7427
Oscillospira	0.72 ± 1.23	0.50 ± 0.70	0.63 ± 0.96	0.5114	0.5076	>0.9999
Faecalibacterium	0.17 ± 0.37	0.16 ± 0.30	0.20 ± 0.46	0.9270	>0.9999	>0.9999
Unclassified Ruminococcaceae	0.39 ± 0.42	0.26 ± 0.33	0.36 ± 0.37	0.4679	0.4961	>0.9999
Unclassified Peptostreptococcus	24.78 ± 16.83	30.62 ± 16.65	26.26 ± 17.91	0.1113	0.0909	>0.9999
[Ruminococcus]	0.79 ± 0.76	0.84 ± 0.66	0.85 ± 0.72	0.8827	>0.9999	>0.9999
Roseburia	0.12 ± 0.19	0.11 ± 0.16	0.08 ± 0.18	0.4225	>0.9999	0.4029
Coprococcus	0.57 ± 1.05	0.48 ± 0.61	0.39 ± 0.50	0.6722	>0.9999	0.7570
Blautia	1.89 ± 1.83	1.66 ± 1.34	1.54 ± 1.14	0.4195	0.9956	0.3850
Unclassified	1.44 ±	1.37 ±	1.44 ±	0.9651	>0.9999	>0.9999

Lachnospiraceae-2	1.53	1.48	1.48			
Unclassified Lachnopiraceae-1	0.31 ± 0.41	0.28 ± 0.37	0.44 ± 0.59	0.2622	>0.9999	0.3848
Pseudoramibacter	0.91 ± 1.16	0.88 ± 1.06	0.71 ± 0.89	0.4375	>0.9999	0.4794
Clostridium	8.68 ± 16.65	6.42 ± 11.03	6.90 ± 13.58	0.4677	0.4463	>0.9999
Unclassified Clostridiaceae	0.26 ± 0.74	0.30 ± 0.81	0.13 ± 0.36	0.2481	>0.9999	0.4366
Unclassifed Clostridiales	0.008 ± 0.016	0.019 ± 0.043	0.006 ± 0.009	0.4757	0.7120	>0.9999
Streptococcus	7.65 ± 14.17	6.84 ± 13.23	6.45 ± 12.30	0.0645	0.2290	0.0457
Enterococcus	7.28 ± 13.22	7.20 ± 11.55	7.41 ± 13.37	0.9787	>0.9999	>0.9999
Parabacteroides	3.81 ± 2.27	3.41 ± 2.47	3.76 ± 2.67	0.3203	0.3353	>0.9999
Bacteroides	10.54 ± 4.22	10.66 ± 4.81	10.96 ± 4.24	0.7798	>0.9999	0.9965
Collinsella	0.49 ± 0.62	0.38 ± 0.46	0.54 ± 0.63	0.1872	0.3981	>0.9999
Bifidobacterium	4.36 ± 5.72	5.44 ± 5.30	5.61 ± 5.85	0.0082	0.0228	0.0082

P value of one-way ANOVA followed by Bonferroni's test on KUHIMM cultures without IgA (Control), cultures with W27 IgA (+ W27 IgA), and cultures with mouse monoclonal IgA (+ Mouse IgA) sampled after 48 h of fermentation from fecal inoculums (n = 12).
